# The pioneer transcription factors Foxa1 and Foxa2 regulate alternative RNA splicing during thymocyte positive selection

**DOI:** 10.1242/dev.199754

**Published:** 2021-07-29

**Authors:** Ching-In Lau, Jasmine Rowell, Diana C. Yanez, Anisha Solanki, Susan Ross, Masahiro Ono, Tessa Crompton

**Affiliations:** 1 UCL Great Ormond Street Institute of Child Health, 30 Guilford Street, London WC1N 1EH, UK; 2 Department of Life Sciences, Imperial College London, London SW7 2AZ, UK

**Keywords:** Foxa1, Foxa2, T-cell development, RNA splicing, Positive selection, Thymus, Mouse

## Abstract

During positive selection at the transition from CD4^+^CD8^+^ double-positive (DP) to single-positive (SP) thymocyte, TCR signalling results in appropriate MHC restriction and signals for survival and progression. We show that the pioneer transcription factors Foxa1 and Foxa2 are required to regulate RNA splicing during positive selection of mouse T cells and that Foxa1 and Foxa2 have overlapping/compensatory roles. Conditional deletion of both Foxa1 and Foxa2 from DP thymocytes reduced positive selection and development of CD4SP, CD8SP and peripheral naïve CD4^+^ T cells. Foxa1 and Foxa2 regulated the expression of many genes encoding splicing factors and regulators, including *Mbnl1*, *H1f0*, *Sf3b1*, *Hnrnpa1*, *Rnpc3*, *Prpf4b*, *Prpf40b* and *Snrpd3*. Within the positively selecting CD69^+^DP cells, alternative RNA splicing was dysregulated in the double Foxa1/Foxa2 conditional knockout, leading to >850 differentially used exons. Many genes important for this stage of T-cell development (*Ikzf1-3*, *Ptprc*, *Stat5a*, *Stat5b*, *Cd28*, *Tcf7*) and splicing factors (*Hnrnpab*, *Hnrnpa2b1*, *Hnrnpu*, *Hnrnpul1*, *Prpf8*) showed multiple differentially used exons. Thus, Foxa1 and Foxa2 are required during positive selection to regulate alternative splicing of genes essential for T-cell development, and, by also regulating splicing of splicing factors, they exert widespread control of alternative splicing.

## Abstract (precis)

**Summary:** The pioneer transcription factors Foxa1 and Foxa2 are required for positive selection during T-cell development in the thymus for regulation of RNA splicing.

## INTRODUCTION

The production of αβ T cells in the thymus involves multiple stages of development during which haematopoietic precursors give rise to mature T cells that can differentiate into functional effector T cells. During this process, progenitors cells that do not express the co-receptor molecules CD4 and CD8 [CD4^−^CD8^−^ double-negative (DN) cells] differentiate to become CD4^+^CD8^+^ double-positive (DP) cells, which give rise to both CD4 single-positive (SP) and CD8SP populations. Maturation from DP to SP follows successful rearrangement of the *Tcra* locus, and requires TCR signalling: positive selection results in appropriate MHC restriction of SP cells, and is followed by negative selection of potentially self-reactive clones and selection of regulatory T cells (Tregs) (Huynh et al., 2014; Littman, 2016; Starr et al., 2003). The strength and duration of the TCR signal that a developing cell receives broadly determine its fate, with the strongest signals leading to negative selection or CD4 Treg differentiation, usually at the SP stage in the medulla, intermediate signals leading to positive selection usually in the cortex, and weaker signals or lack of TCR signalling leading to death by neglect (Singer et al., 2008). For DP thymocytes undergoing positive selection, TCR signal strength and duration also influence CD4 and CD8 lineage choice. Those cells receiving stronger and longer TCR signals tend towards the CD4SP fate, whereas weaker/more transient signals favour the CD8SP fate, and fate decisions are also influenced by the relative timing of cytokine and TCR signalling that a developing cell receives (Littman, 2016; Klein et al., 2014; Bosselut, 2004). Many models have been proposed to describe this process and to explain how positive selection ensures that CD4SP and CD8SP populations express TCR appropriately restricted by MHCII and MHCI, respectively (Littman, 2016; Starr et al., 2003; Carpenter and Bosselut, 2010). Currently, the consensus favours the kinetic signalling model (Littman, 2016; Singer et al., 2008; Egawa, 2015), in which CD8 is downregulated first during positive selection, leading to a CD4^+^CD8^lo^ intermediate, with continued CD4 co-receptor expression allowing for prolonged stronger MHCII-TCR signalling, leading to differentiation to CD4SP, whereas cytokine signalling through the common gamma chain activates Stat5a and Stat5b and rescues cells that have received an interrupted MHCI-TCR signal to induce differentiation to CD8SP (Park et al., 2010; Brugnera et al., 2000). The CD4/CD8 lineage decision is also influenced by factors from the stroma, such as Notch and Hedgehog (Hh) signalling (Laky and Fowlkes, 2008; Solanki et al., 2018; Furmanski et al., 2012; Rowbotham et al., 2007).

Many transcription factors contribute to regulation of the CD4/CD8 lineage decision (Littman, 2016; Carpenter and Bosselut, 2010; Taniuchi, 2016; Naito et al., 2011). Additionally, epigenetic processes, such as DNA methylation and histone modification, may be involved in ‘locking-in’ the pattern of gene expression to generate stable CD4SP and CD8SP lineages (Issuree et al., 2017), and potentially also in preparing for initiation of a particular programme of differentiation.

Here, we investigate the role of the transcription factors Foxa1 and Foxa2 in T-cell development. The Foxa proteins are a highly conserved subfamily of forkhead box transcription factors, which contain unique wing-helix DNA-binding domains (Jackson et al., 2010). The Foxa proteins can function as pioneer transcription factors, which by binding silent (condensed) chromatin early in a developmental programme prior to target gene activation, can act either to open up local chromatin, imparting competence to other transcriptional activators to initiate a developmental lineage or to directly facilitate other factors binding to nucleosomal DNA (Iwafuchi-Doi et al., 2016; Zaret, 2020). Foxa2 has recently also been shown to demethylate tissue-specific regions of DNA, to generate stable lineage-specific DNA methylation patterns that enhance gene expression (Reizel et al., 2021).

Foxa1 and Foxa2 proteins are closely related to each other and are widely co-expressed during embryogenesis and in several tissues postnatally, including lung, liver, intestines, pancreas and thymus (Solanki et al., 2018; Kaestner, 2010; Besnard et al., 2004; Kaestner et al., 1994; Lau et al., 2018; Rowbotham et al., 2009). Genetic ablation of Foxa1 or Foxa2 in mice showed that they are both required for normal development during embryogenesis. Foxa2-deficient embryos display severe defects in notochord, floorplate and endoderm and die at embryonic day (E) 10-11, whereas Foxa1-null mice exhibit defects in the regulation of glucose homeostasis and die postnatally (Weinstein et al., 1994; Shih et al., 1999). Foxa1 and Foxa2 play overlapping and compensatory roles in the regulation of development of lung, liver and pancreas (Kaestner, 2010; Gao et al., 2008; Reizel et al., 2020; Lee et al., 2005; Wan et al., 2005).

Foxa2 is a well-recognized target gene of the Shh signalling pathway in the floorplate, and is required for the maintenance of Shh expression, but can also negatively regulate expression of components of the Shh signalling pathway (Sasaki et al., 1997; Metzakopian et al., 2012; Jeong and Epstein, 2003). Expression of Foxa1 is also correlated with Hh signalling activities, and Foxa1-deficient mice exhibited elevated Shh and Gli2 expression in prostate, suggesting that Foxa1 can negatively regulate Shh signalling (Gao et al., 2005).

Little is known about the function of Foxa1 and Foxa2 in the immune system. In mouse models of autoimmune inflammation, ectopic Foxa1 expression has been shown to drive the differentiation and suppressive function of a novel subset of Tregs (Liu et al., 2014). We recently found that expression of Foxa1 and Foxa2 in thymic epithelial cells (TECs) is required to maintain normal T-cell development and homeostasis of thymic and spleen Treg populations. Conditional deletion of Foxa1 and Foxa2 from TECs increased the proportion of medullary TECs, but reduced cell-surface MHCII expression on TECs, leading to a smaller thymus with a reduction in conventional CD4 T-cell differentiation but an increase in the CD4 Treg population (Lau et al., 2018). In the thymus, Foxa1 and Foxa2 are also expressed in developing T cells, and Foxa2 is a transcriptional target of Shh signalling after pre-TCR signal transduction (Solanki et al., 2018; Rowbotham et al., 2009).

Here, we investigate the function of Foxa1 and Foxa2 in developing αβT cells. We show that Foxa1 and Foxa2 are required at the transition from DP to SP T cell by regulation of RNA splicing. Conditional deletion of both Foxa1 and Foxa2 led to a reduction in positive selection, differentiation and maturation of the SP populations, and a reduction in the peripheral CD4 T-cell pool.

## RESULTS

### CD4SP and CD8SP development are impaired in the *Foxa1/2* conditional knockout thymus

**Fig. 1.**
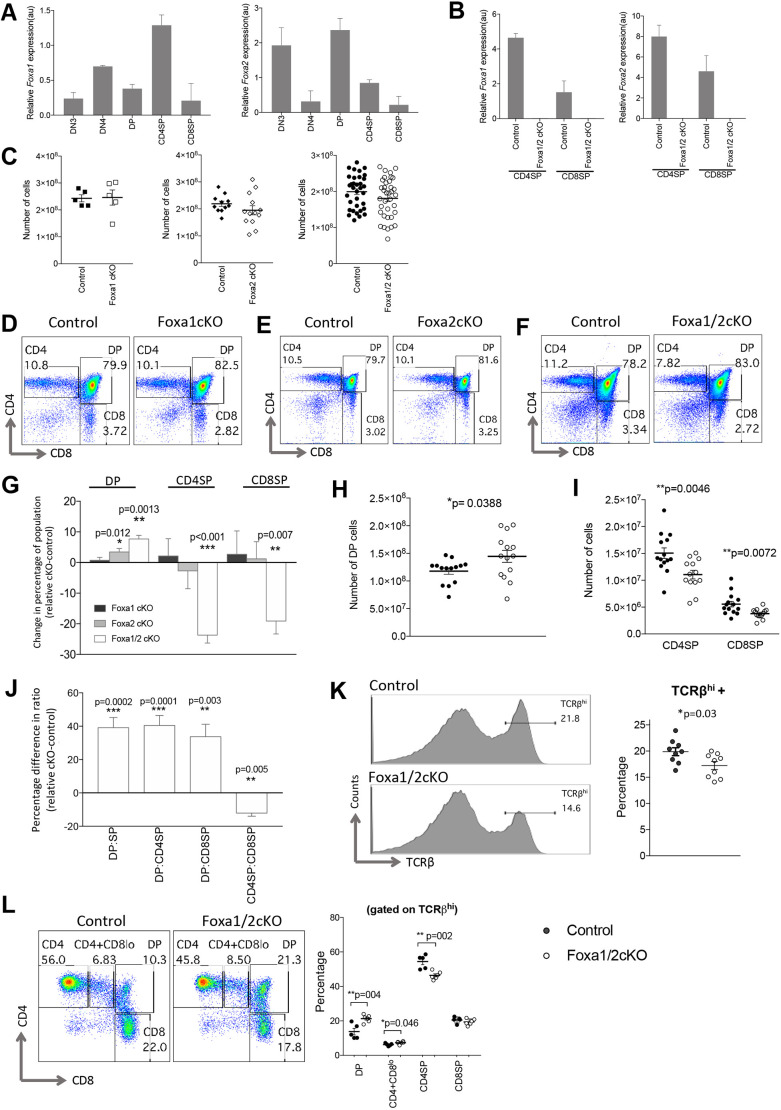
**Foxa1 and Foxa2 are expressed in thymocytes and required in T-cell development.** (A) *Foxa1* and *Foxa2* expression (relative to *Gapdh*) were measured by qRT-PCR in FACS-sorted thymocyte populations from wild-type (C57BL/6) adult mice: DN3 (CD4^−^CD8^−^CD25^+^CD44^−^); DN4 (CD4^−^CD8^−^CD25^−^CD44^−^); DP (CD4^+^CD8^+^); CD4SP (CD4^+^CD8^−^CD3^+^); CD8SP (CD4^−^CD8^+^CD3^+^). Bar charts show relative expression of *Foxa1* (left) and *Foxa2* (right) in these FACS-sorted populations. (B) Bar charts show relative expression of *Foxa1* (left) and *Foxa2* (right) (relative to *Gapdh*) measured by qRT-PCR in FACS-sorted CD4SP (CD4^+^CD8^−^CD3^+^) and CD8SP (CD4^−^CD8^+^CD3^+^) cells from Foxa1/2cKO and control thymus. (C) Scatter plots show the number of thymocytes recovered from the thymus of Foxa1cKO, Foxa2cKO and Foxa1/2cKO mice compared with control littermates. (D-F) Flow cytometry profiles show anti-CD4 and anti-CD8 staining, giving the percentage of cells in the region shown for Foxa1cKO (D), Foxa2cKO (E) and Foxa1/2cKO (F) thymus compared with control littermate thymus. (G) Bar chart shows the change in the percentage of the thymocyte population in cKO relative to control littermates (mean percentage of each population in control littermates subtracted from relative percentage of same population in conditional knockout) in Foxa1cKO, Foxa2cKO and Foxa1/2cKO thymus. For control versus Foxa1cKO: control *n*=5, Foxa1cKO *n*=5; for control versus Foxa2cKO: control *n*=9, Foxa1cKO *n*=9; for control versus Foxa1/2cKO: control *n*=15, Foxa1/2cKO *n*=16. (H,I) Scatter plots show the number of cells in DP (H) and CD4SP and CD8SP (I) populations in Foxa1/2cKO mice compared with control. (J) Bar chart shows the change in thymocyte subset ratio in Foxa1/2cKO relative to control littermates, calculated by the mean ratio of control littermates subtracted from the relative ratio from Foxa1/2cKO; control *n*=15, Foxa1/2cKO *n*=16. (K) Histograms show staining of anti-TCRβ on thymocytes, giving the percentage of TCRβ^hi^ cells in the marker shown (TCRβ^hi^). Scatter plot shows the percentage of TCRβ^hi^ cells in control and Foxa1/2cKO littermates. Horizontal bars indicate mean. (L) Flow cytometry profiles show anti-CD4 and anti-CD8 staining of thymus, gated on the TCRβ^hi^ population, giving the percentage of cells in the regions shown. Scatter plot shows the percentage of DP, CD4^+^CD8^lo^, CD4SP and CD8SP, gated on TCRβ^hi^ in Foxa1/2cKO and control thymus. In scatter plots, each symbol represents an individual mouse, either control (black circles) or Foxa1/2cKO (white circles). Bar charts and scatter plots show mean and s.e.m., giving significance by Student's *t*-test: *P<0.05; **P<0.01; ***P<0.001. au, arbitrary units.

We examined *Foxa1* and *Foxa2* expression by qRT-PCR in FACS-sorted developing thymocytes from the DN3 stage onwards (Fig. 1A). *Foxa1* and *Foxa2* were detected at all stages, and showed reciprocal patterns of expression, with *Foxa1* expression upregulated in DN4, followed by a decline in DP cells. In contrast, *Foxa2* was highly expressed at DN3 and DP stages, and expressed at lower levels in DN4 cells. Both *Foxa1* and *Foxa2* showed higher expression in CD4SP than in CD8SP cells.

To establish whether Foxa1 and Foxa2 play a T-cell intrinsic role in thymocyte development, we conditionally deleted *Foxa1* and/or *Foxa2* from T cells from the DP stage of development onwards, by crossing mice carrying a single or double loxP-flanked *Foxa1* and/or *Foxa2* allele to mice in which Cre is driven by the *Cd4* promotor. Foxa1^fl/fl^CD4cre^+^ and Foxa2^fl/fl^CD4cre^+^ mutant mice are referred to as Foxa1cKO and Foxa2cKO, respectively. Foxa1^fl/fl^Foxa2^fl/fl^CD4cre^+^ double mutant mice are referred to as Foxa1/2cKO. *Foxa1* and *Foxa2* were effectively deleted in Foxa1/2cKO CD4SP and CD8SP thymocytes, as expression of *Foxa1* and *Foxa2* were below detection by qRT-PCR, but detected in the control (Foxa1^fl/fl^Foxa2^fl/fl^CD4cre^−^) CD4SP and CD8SP populations (Fig. 1B).

We compared thymocyte populations in Foxa1cKO, Foxa2cKO and Foxa1/2cKO thymus with their control littermates. The number of thymocytes were not significantly different between the conditional Foxa1 and/or Foxa2 mutants and their controls (Fig. 1C). The Foxa1cKO thymus contained normal proportions of DP and SP populations (Fig. 1D), but there was an increase in the proportion of DP cells in the Foxa2cKO compared with control (Fig. 1E,G). In the Foxa1/2cKO thymus, the proportion and number of the DP population were increased and the proportion and number of CD4SP and CD8SP populations were reduced (Fig. 1F-I). The fact that, although the phenotype of the double Foxa1/2cKO thymus was more pronounced than that of the Foxa2cKO, the Foxa1cKO thymus appeared grossly normal, suggests that Foxa1 can compensate for Foxa2 at this developmental transition, and we therefore decided to use the double Foxa1/2cKO mice to investigate the impairment of differentiation from DP to SP cell.

The ratios of DP:SP, DP:CD4SP and DP:CD8SP were all increased in Foxa1/2cKO compared with control, whereas the ratio of CD4SP:CD8SP was decreased (Fig. 1J). Thus, deficiency of both Foxa1 and Foxa2 in thymocytes led to less efficient development of SP populations and bias towards the generation of CD8 lineage cells over CD4SP. During differentiation from DP to SP stage, cell surface TCR expression is upregulated. The proportion of TCRβ^hi^ thymocytes was decreased in the absence of Foxa1 and Foxa2 (Fig. 1K). When we gated on TCRβ^hi^ cells, and compared subset distribution, we found an increase in the proportion of DP and CD4^+^CD8^lo^ intermediate cells, and a decrease in the proportion of CD4SP population, indicating that fewer cells were completing positive selection and a partial arrest at the transition from DP and CD4^+^CD8^lo^ to CD4SP (Fig. 1L).

### Foxa1/2 deficiency influences maturation of SP cells and peripheral T-cell populations

**Fig. 2.**
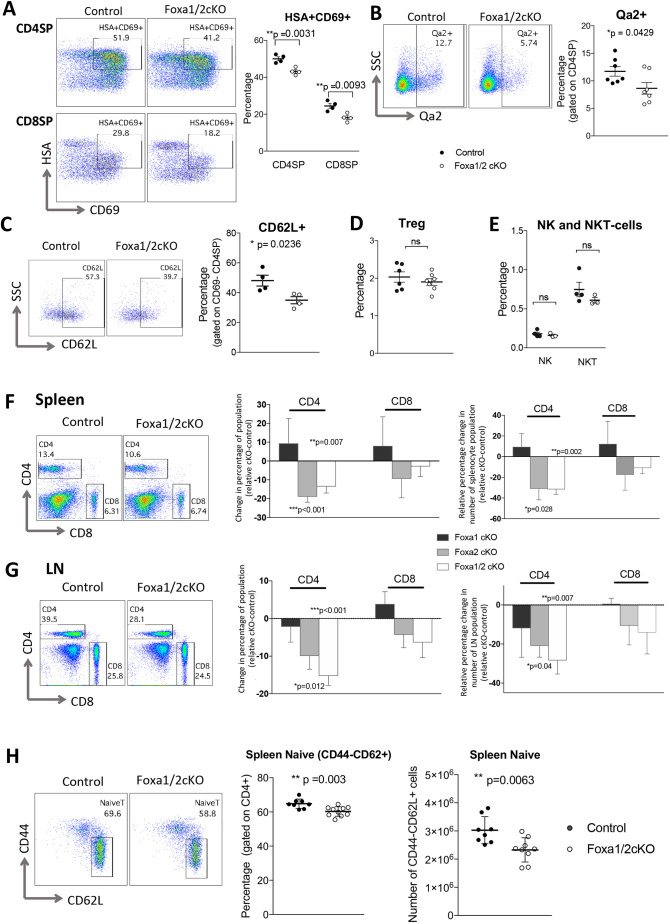
**Maturation of SP and peripheral T cells in Foxa1/2cKO mice.** (A) Flow cytometry profiles show anti-HSA and anti-CD69 staining on CD4SP and CD8SP cells, giving the percentage of cells in the region shown. Scatter plot shows the percentage of HSA^+^CD69^+^ cells in the CD4SP and CD8SP populations for control and Foxa1/2cKO. (B) Flow cytometry profiles show anti-Qa2 staining, gated on CD4SP, giving the percentage of cells in the region shown. Scatter plot shows percentage of Qa2^+^ cells in the CD4SP population. (C) Flow cytometry profiles show anti-CD62L staining, gated on the CD69^−^CD4SP population. Scatter plot shows the percentage of CD62L^+^ cells in the CD69^−^CD4SP population. (D) Percentage of Tregs (CD8^−^CD4^+^CD25^+^icFoxp3^+^) in the control and Foxa1/2cKO thymus. (E) Percentage of NK (CD3^−^NK1.1^+^) and NKT cells (CD3^+^NK1.1^+^) in the control and Foxa1/2cKO thymus. (F,G) Flow cytometry profiles show anti-CD4 and anti-CD8 staining of spleen (F) and lymph node (LN) (G) in Foxa1/2cKO and control mice. Bar charts illustrate the percentage change in T-cell composition and actual cell numbers in conditional knockout mice relative to control littermates in spleen (F) and LN (G). Differences in percentage were calculated by subtracting the mean percentage or population number in control littermates from the relative percentage or population number in cKO. (H) Flow cytometry profiles show anti-CD44 and anti-CD62L staining, gated on CD4^+^ T cells in Foxa1/2cKO and control spleen, giving the percentage of cells in the region shown. Scatter plots show the percentage of CD44^−^CD62L^+^ cells (gated on CD4^+^) and number of CD44^−^CD62L^+^ CD4^+^ T cells (naïve T cells) in Foxa1/2cKO and control spleen. In scatter plots, each symbol represents an individual mouse, either control (black circles) or Foxa1/2cKO (white circles). Scatter plots and bar charts show mean and s.e.m., giving significance by Student's *t*-test: *P<0.05; **P<0.01; ***P<0.001. ns, not significant.

We then investigated the maturation status of SP cells in the Foxa1/2cKO thymus by expression of heat-stable antigen (HSA; CD24), CD69, Qa2 and CD62L. After positive selection, SP thymocytes retain high expression of HSA and CD69, and as they mature HSA and CD69 are downregulated, Qa2 is upregulated, and expression of CD62L indicates that thymocytes are mature and ready to egress from the thymus. The proportion of HSA^+^CD69^+^ cells in both CD4SP and CD8SP populations was significantly decreased in Foxa1/2cKO compared with control thymus (Fig. 2A). Furthermore, there was a significant decrease in the percentage of Qa2^+^ and CD69^−^CD62L^+^ cells in the CD4SP compartment in Foxa1/2cKO compared with control (Fig. 2B,C), whereas we did not detect significant differences in Qa2 and CD62L expression in the CD8SP population (Fig. S2). Thus, in the absence of Foxa1 and Foxa2, not only were there fewer CD4SP and CD8SP cells, but their maturation was also affected, such that fewer mature CD62L^+^CD4SP were produced to egress from the thymus. In contrast, we found no significant difference in the proportion of CD4^+^CD25^+^Foxp3^+^ Tregs, NKT cells or NK cells in the Foxa1/2cKO thymus compared with control (Fig. 2D,E).

In the spleen and lymph nodes, changes in the CD4^+^ T-cell populations mirrored the thymus in the Foxa1/2cKO compared with control, with reductions in the proportion and number of conventional CD4^+^ T cells overall and of naïve CD44^−^CD62L^+^CD4^+^ T cells in spleen (Fig. 2F-H). In contrast, we did not detect significant differences in the number of peripheral CD8^+^ T cells in the Foxa1/2cKO compared with control, suggesting that, despite the reduction in CD8SP cells in the thymus, the peripheral CD8^+^ T-cell compartment is subject to homeostatic control and can expand to reach its normal size (Fig. 2F,G).

### Foxa1/2 deficiency reduces TCR signalling

**Fig. 3.**
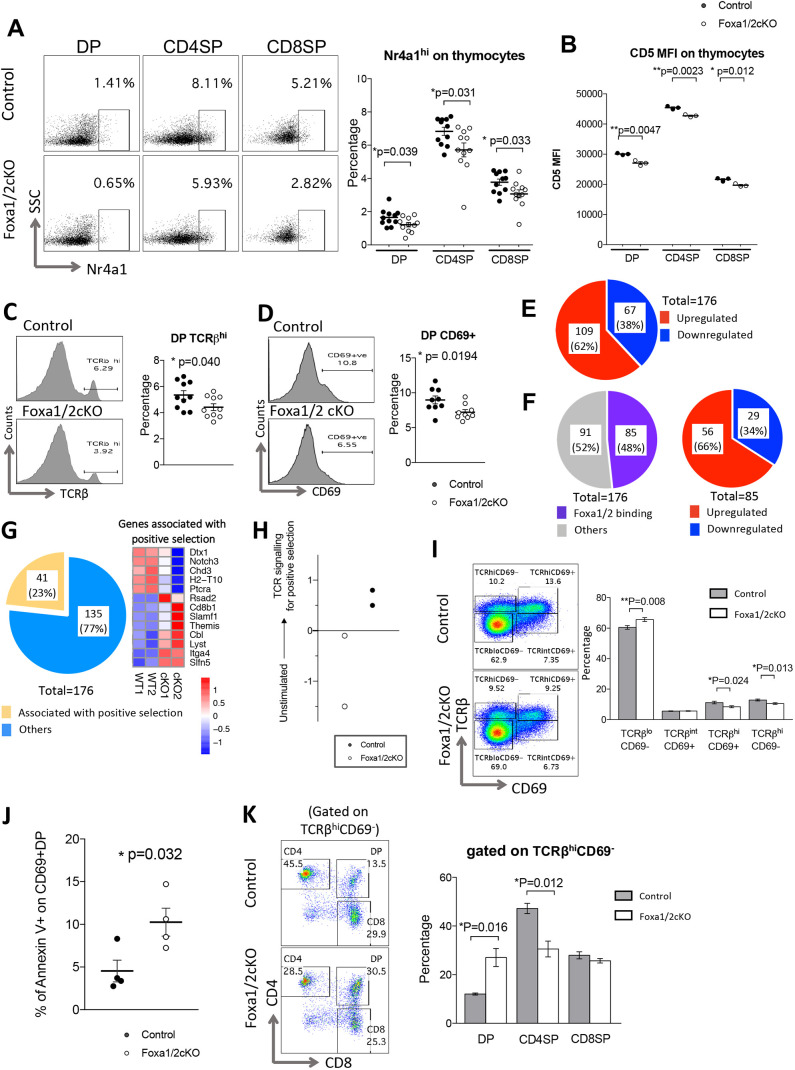
**Foxa1/2 regulates TCR signal and promotes positive T-cell selection.** (A) Flow cytometry profiles show intracellular anti-Nr4a1 staining, gated on DP, CD4SP and CD8SP in control and Foxa1/2cKO thymus. Scatter plot shows percentage of Nr4a1^hi^ in DP, CD4SP and CD8SP populations. (B) Scatter plot shows MFI of anti-CD5 staining on DP, CD4SP and CD8SP populations. (C,D) Histograms show staining of anti-TCRβ (C) and anti-CD69 (D) on the DP population in control and Foxa1/2cKO thymus. Scatter plots show percentage of TCRβ^hi^ (C) and CD69^+^ (D) on DP populations. (E) Pie chart represents the proportion of DEGs that were upregulated (red) or downregulated (blue) in Foxa1/2cKO datasets compared with control. (F) Left: Pie chart illustrates number of DEGs that were identified as binding Foxa1/2 in genome-wide ChipSeq analysis of Foxa1/2 binding sites in neuronal progenitors (Metzakopian et al., 2012, 2015) (purple) and DEGs that were not (grey). Right: Pie chart represents the 85 DEGs that were found to be Foxa1/2-binding, as defined in F, that were upregulated (red) or downregulated (blue) in our Foxa1/2cKO datasets. (G) Pie chart represents the number of DEGs that are associated with positive selection (Kasler et al., 2011) (yellow) and that are not associated with positive selection (blue). Pearson correlation clustering heatmap shows expression of selected positive selection-associated DEGs in control and Foxa1/2cKO, where red represents higher expression and blue lower expression on a linear correlation scale. A value of 1 indicates a positive association, a value of −1 indicates a negative association, and a value of 0 indicates no association. (H) Scatter plot shows canonical correspondence analysis on a scale of unstimulated to TCR-signalling-for positive-selection (Lo et al., 2012). (I) Flow cytometry profiles show anti-TCRβ and anti-CD69 staining, giving the percentage in regions shown for TCRβ^lo^CD69^−^ (pre-selection), TCRβ^int^CD69^+^ (selecting), TCRβ^hi^CD69^+^ (post initiation of positive selection) and TCRβ^hi^CD69^−^ (mature). Histogram shows the mean percentage of cells in these four subsets, defined by staining against TCRβ and CD69. (J) Scatter plot shows the percentage of annexin V^+^ cells in the CD69^+^DP population. (K) Flow cytometry profiles show anti-CD4 and anti-CD8 staining gated on the TCRβ^hi^CD69^−^ population. Histogram shows the mean percentage of each thymocyte subpopulation, gated on TCRβ^hi^CD69^−^. In scatter plots, each symbol represents an individual mouse, either control (black circles) or Foxa1/2cKO (white circles). Scatter plots and bar charts show mean and s.e.m., giving significance by Student's *t*-test: *P<0.05; **P<0.01.

TCR signal strength is one factor that determines positive selection in the thymus, and tonic TCR signalling is again required for maintenance and homeostasis of peripheral T-cell populations after egress from the thymus. Therefore, as a proxy to measure TCR signal strength in thymocyte subsets, we compared the expression of proteins for which expression levels are directly determined by TCR signalling between Foxa1/2cKO and control. Expression of intracellular Nr4a1 is induced as an early consequence of TCR signal transduction and high expression requires relatively strong TCR signalling (Moran et al., 2011; Bending et al., 2018). The proportion of intracellular Nr4a1^hi^ cells was significantly reduced in Foxa1/2cKO DP, CD4SP and CD8SP populations compared with control, suggesting that in the absence of Foxa1 and Foxa2 fewer cells had reached the threshold of TCR signal strength required to induce high levels of Nr4a1 (Fig. 3A). Levels of cell-surface CD5 expression correlate with the strength of TCR signal transduction that a developing T cell has received (Azzam et al., 2001). As expected, mean fluorescence intensity (MFI) of anti-CD5 staining was lower in CD8SP and DP cells than in CD4SP cells (Fig. 3B). MFI of CD5 was lower in Foxa1/2cKO DP, CD4SP and CD8SP populations than in their control counterparts, consistent with reduced TCR signal strength in the absence of Foxa1 and Foxa2.

### Foxa1 and Foxa2 promote positive selection in DP thymocytes

Positive selection signals for survival and further maturation and requires interactions between self-peptide:MHC complexes in the thymic cortex, which may take place over a number of days, involving multiple or prolonged TCR-MHC interactions (Ross et al., 2014; Kisielow and Miazek, 1995; Liu and Bosselut, 2004). TCR signalling for positive selection leads to cell-surface CD69 expression followed by upregulation of the cell-surface TCR complex. The proportions of DP cells that expressed cell surface TCRβ^hi^ and CD69 were decreased in the Foxa1/2cKO thymus compared with control (Fig. 3C,D), consistent with a reduction in positive selection. To test this and to investigate mechanisms that might account for the reduced transition from DP to SP, we carried out transcriptome analysis on cells undergoing positive selection by RNA sequencing FACS-sorted CD69^+^DP cells. RNA sequencing identified only 176 differentially expressed genes (DEGs) between Foxa1/2cKO and control datasets (FDR-adjusted *P*<0.05), of which 109 (62%) were more highly expressed in Foxa1/2cKO than in control (Fig. 3E, Table S1). Of the 176 DEGs, 85 (∼48%) had previously been identified as Foxa1/2 targets in genome-wide ChipSeq analysis of Foxa1/2-binding sites in dopaminergic neuronal progenitors and 56 of those genes with verified Foxa1/2-binding sites (approximately two-thirds) showed higher expression in the Foxa1/2cKO than in control (Fig. 3F, Table S1) (Metzakopian et al., 2012, 2015). DEGs included genes involved in T-cell development and function, and approximately one-quarter of DEGs were genes for which transcription has been shown to be regulated during positive selection (Kasler et al., 2011) (Fig. 3G, Table S1). Among these, DEGs that were more highly expressed in Foxa1/2cKO than in control datasets included genes known to reduce TCR signal strength [*Cbl*, a ubiquitin ligase that negatively regulates TCR signalling (Huang and Gu, 2008), and *Themis*, which can attenuate TCR signalling during repertoire selection (Gascoigne and Acuto, 2015)] and genes associated with the CD8 lineage [*Cd8b1* and *Lyst*, a lysosomal trafficking regulator required for CTL lytic granules (Sepulveda et al., 2015)], consistent with the CD4 lineage being more severely affected in the conditional knockouts.

To test in an unbiased way whether Foxa1 and Foxa2 are required for the transcriptional response to TCR signalling for positive selection, we then used canonical correspondence analysis (CCA; see Materials and Methods) to compare the overall pattern of gene expression in our datasets with transcriptome data from publicly available datasets prepared from DP thymocytes that were receiving different strengths of TCR signals during selection (GSE38909) (Lo et al., 2012). The GSE38909 dataset contains DP thymocytes from AND-TCR transgenic mice stimulated with a positively selecting peptide (gp250) or a non-selecting control peptide (Lo et al., 2012). We selected the 2000 genes that were most significantly differentially expressed from the GSE38909 dataset between DP thymocytes stimulated with the non-selecting control peptide and DP thymocytes stimulated with the positively selecting peptide and used these to generate a scale of unstimulated to TCR-signalling-for-positive-selection. We plotted our datasets against this scale. The CCA segregated the datasets by genotype: both control datasets were on the positive side of the axis, which corresponded to the transcriptional pattern induced by the positively selecting peptide, consistent with the fact that CD69^+^DP thymocytes have initiated positive selection by TCR signal transduction (Fig. 3H). In contrast, both Foxa1/2cKO datasets fell on the negative side of the axis, thus showing an overall pattern of transcription that is closer to that of unstimulated DP cells than that of their control counterparts.

To confirm this impact on positive selection, we subdivided thymocytes by cell surface TCRβ and CD69 expression into four different stages: TCRβ^lo/neg^CD69^−^ (pre-selection thymocytes), TCRβ^int^CD69^+^ (intermediate transition, undergoing positive selection), TCRβ^hi^CD69^+^ (thymocytes after TCR signalling for initiation of positive selection) and TCRβ^hi^CD69^−^ (more mature population). The proportion of pre-selection thymocytes was higher in the Foxa1/2cKO than control thymus, whereas there was no significant difference in the proportion of the TCRβ^int^CD69^+^ population, and both TCRβ^hi^CD69^+^ and TCRβ^hi^CD69^−^ populations were reduced, indicating that Foxa1 and Foxa2 expression in thymocytes promotes initiation of the process of positive selection, and also progression of cells during the differentiation process (Fig. 3I). The loss of cells from the TCRβ^hi^CD69^+^ population, but not its precursor TCRβ^int^CD69^+^ population, suggests impairment at a late stage of positive selection and a failure to progress, and, consistent with this, the proportion of cells undergoing cell death (annexin V^+^) was increased in the CD69^+^DP population in the Foxa1/2cKO thymus compared with control (Fig. 3J). When we gated on the most mature TCRβ^hi^CD69^−^ thymocyte population, and compared CD4/CD8 subset distribution, we found that the proportion of DP cells increased by more than two-fold in the Foxa1/2cKO thymus compared with control, whereas the proportion of CD4SP cells was significantly reduced (Fig. 3K), demonstrating a clear requirement for Foxa1 and Foxa2 in normal differentiation to CD4SP and positive selection, and suggesting that differentiation is dysregulated in the Foxa1/2cKO, so that more cells that had upregulated cell surface TCR and downregulated CD69 were unable to progress beyond the DP stage.

### Foxa1 and Foxa2 regulate exon usage

**Fig. 4.**
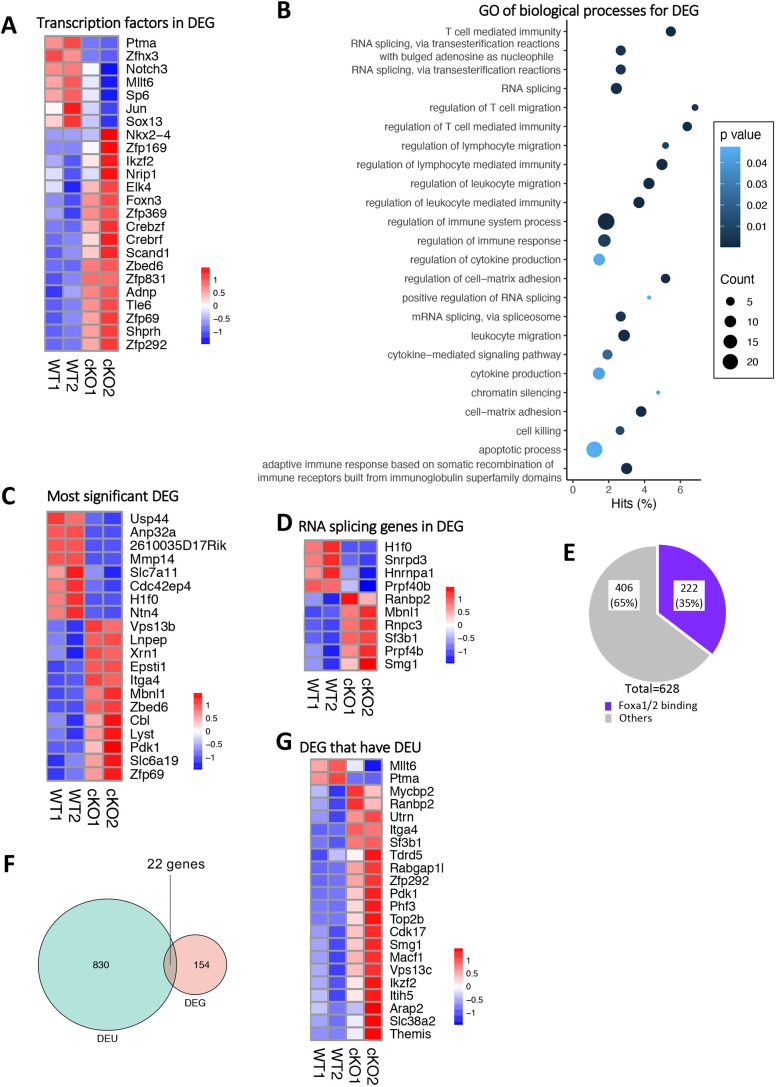
**Foxa1/2 deficiency disturbs exon usage in developing T cells.** (A) Pearson correlation clustering heatmap shows expression of DEGs that encode transcription factors in control and Foxa1/2cKO, where red represents higher expression and blue lower expression on a linear correlation scale. A value of 1 indicates a positive association, a value of −1 indicates a negative association, and a value of 0 indicates no association. (B) Dot plot of Gene Ontology (GO) analysis of biological processes associated with 176 DEGs. The *y*-axis represents the biological process GO terms and the *x*-axis represents the percentage of genes found in that GO term category. Dot size represents the number of genes and the colour indicates the *P*-value. (C) Pearson correlation clustering heatmap shows expression of the 20 most significant DEGs in control and Foxa1/2cKO, where red represents higher expression and blue lower expression on a linear correlation scale. A value of 1 indicates a positive association, a value of −1 indicates a negative association, and a value of 0 indicates no association. (D) Pearson correlation clustering heatmap shows expression of DEGs associated with RNA splicing in control and Foxa1/2cKO, where red represents higher expression and blue lower expression on a linear correlation scale. A value of 1 indicates a positive association, a value of −1 indicates a negative association, and a value of 0 indicates no association. (E) Pie chart illustrates the number of genes showing DEU: purple section represents the number of genes that are identified as Foxa1/2 targets in genome-wide ChipSeq analysis of Foxa1/2-binding sites in neuronal progenitors (Metzakopian et al., 2012, 2015). (F) Proportional Venn diagram illustrates the intersection between DEGs and genes showing DEU. (G) Pearson correlation clustering heatmap shows expression of the 22 DEGs that show DEU, where red represents higher expression and blue lower expression on a linear correlation scale. A value of 1 indicates a positive association, a value of −1 indicates a negative association, and a value of 0 indicates no association.

To investigate how Foxa1/2 might regulate positive selection, we identified genes that encode verified transcription factors among the DEGs between Foxa1/2cKO and control CD69^+^DP datasets to look for known regulators of positive selection or differentiation at this developmental stage that might function downstream of Foxa1/2. Twenty-four transcription factors were found, of which 13 had previously been verified as Foxa1/2 targets in genome-wide ChipSeq analysis in neuronal progenitors (Metzakopian et al., 2012, 2015) (Fig. 4A, Table S1). Among the transcription factors for which expression was downregulated by Foxa1/2 deletion, there were no obvious downstream candidates that might function to promote positive selection, although several showed changes in expression consistent with the developmental phenotype, for example *Ptma*, an anti-apoptotic gene (Jiang et al., 2003), and *Notch3*, which is transcriptionally regulated during positive selection but not required for positive selection (Kasler et al., 2011; Suliman et al., 2011). Likewise, we found no obvious candidate genes that might function to inhibit positive selection or TCR signal strength among those genes that encode transcription factors and for which expression was upregulated in the absence of Foxa1/2. *Ikzf2* was more highly expressed in Foxa1/2cKO than in control, but is not required for differentiation at this stage of development; *Elk4* was also more highly expressed, but is required for positive selection and upregulated by TCR signal transduction, rather than functioning as a negative regulator (Bending et al., 2018; Georgopoulos, 2017; Costello et al., 2004; Wang et al., 2010).

We then carried out Gene Ontology (GO) term enrichment analysis of all DEGs between Foxa1/2cKO and control CD69^+^DP datasets. This revealed over-representation of genes associated with terms connected with immunity and T cell-mediated immunity, including lymphocyte migration, cytokine signalling, cell killing and the apoptotic process (Fig. 4B, Table S2). Thus, as well as an overall enrichment in immunity-related terms, as expected from the cell type, there was enrichment for terms related to processes required for differentiation to SP, such as lymphocyte migration, as differentiating cells must migrate to the medulla to complete their maturation and repertoire selection. Several terms related to cytokine signalling are also pertinent to the partial arrest at the DP and CD4^+^CD8^lo^ stage in the Foxa1/2cKO, as cytokine signalling rescues cells for which the MHCI-restricted TCR signal has been interrupted by downregulation of CD8, allowing differentiation to CD8SP, whereas cytokine signalling is inhibited for differentiation to the CD4SP compartment.

Additionally, there was enrichment for the term ‘chromatin silencing’, related to the known function of Foxa transcription factors as pioneer factors. The GO analysis also showed enrichment for several terms related to RNA splicing, and for terms connected to cell matrix adhesion (Fig. 4B). Among the top 20 most statistically significant DEGs were genes associated with these processes (Fig. 4C), for example, *Mmp14*, a membrane-bound matrix metalloproteinase involved in breakdown of the extracellular matrix (Kessenbrock et al., 2010); *H1f0*, an H1 linker histone necessary for the condensation of nucleosome chains into higher-order chromatin structures, which is involved in the regulation of mRNA splice site recognition (Kalashnikova et al., 2013; Di Liegro et al., 2018); and *Mbnl1*, a regulator of alternative splicing that functions in the control of T-cell development, and is overexpressed in mixed lineage leukaemia (Ho et al., 2004; Wang et al., 2012; Sznajder et al., 2020; Itskovich et al., 2020).

Several other RNA-splicing genes were differentially expressed between Foxa1/2cKO and control datasets (Fig. 4D). Given this, and the fact that the GO enrichment analysis identified several terms associated with RNA splicing, we hypothesized that Foxa1/2 might regulate the transition from DP to SP cell and positive selection by influencing RNA splicing in developing T cells. To test this, we compared differential exon usage (DEU) between Foxa1/2cKO and control CD69^+^DP RNA-sequencing datasets. This analysis identified 852 events of DEU (FDR *P*<0.05) between the conditional knockout and control, which involved 628 different genes, 222 (∼35%) of which had previously been shown to bind to Foxa1/2 in genome-wide ChipSeq analysis of Foxa1/2-binding sites in dopaminergic neuronal progenitors (Fig. 4E, Table S3) (Metzakopian et al., 2012, 2015). Conditional deletion of Foxa1/2 therefore led to a greater number of changes in exon usage affecting more genes than the number of individual genes that were differentially expressed. Intersection between DEGs and genes that contained differentially used exons revealed just 22 DEGs (overall expression of which was differentially regulated and which also showed differential usage of individual exons) (Fig. 4F), 18 of which (>80%) had previously verified Foxa1/2-binding sites (Table S1). This intersection included several genes involved in the regulation of RNA splicing, with known splicing variants (Fig. 4G): *Ranbp2*, a nucleoporin protein that controls alternative-splicing patterns during nuclear speckle formation (Saitoh et al., 2012); *Sf3b1*, a well-known splicing factor, mutations of which lead to myelodysplasia and anaemia by globally disrupted splicing (Shiozawa et al., 2018; Mupo et al., 2017); and *Smg1*, which is mutated in acute myeloid leukaemia, its depletion resulting in disruption of alternative splicing (McIlwain et al., 2010; Du et al., 2014).

**Fig. 5.**
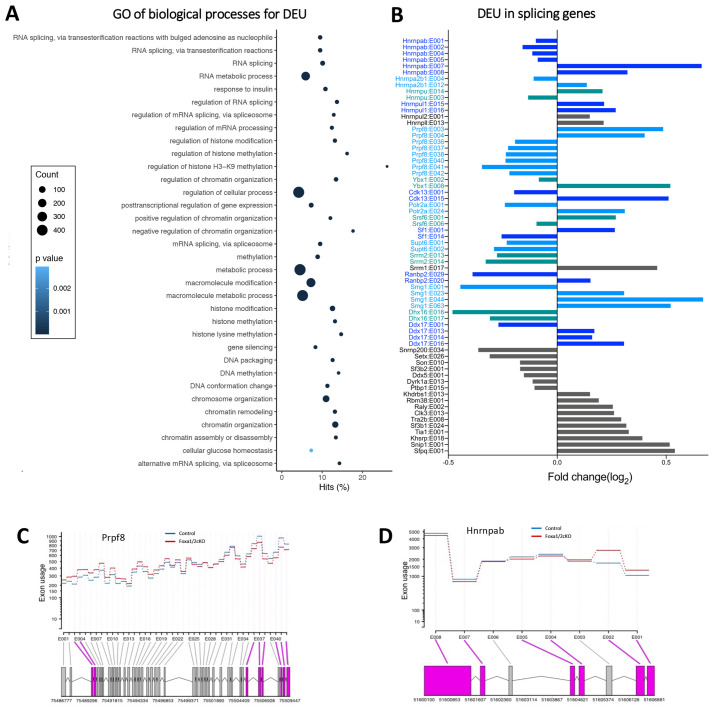
**Foxa1/2 regulate alternative splicing of splicing factors.** (A) Dot plot of Gene Ontology (GO) analysis for genes showing DEU. Biological processes involving pioneer function, methylation, metabolic processes and RNA splicing are shown. The *y*-axis represents the biological process GO terms and the *x*-axis represents the percentage of genes found in that GO term category. Dot size represents the number of genes and the colour indicates the *P*-value. (B) Bar chart shows genes that contain DEU which are involved in mRNA splicing. Genes shown in colour show multiple DEU, genes in grey show single differential exon changes. (C,D) DEXSeq representations of *Prpf8* (C) and *Hnrnpab* (D) that show significant DEU between Foxa1/2cKO (red) in comparison with control (blue). The exons highlighted in pink have DEU (FDR-adjusted *P*≤0.05).

GO term enrichment analysis of genes that showed DEU revealed over-representation of genes associated with terms connected with the known functions of Foxa1/2 in metabolic processes (‘metabolic process’, ‘cellular glucose homeostasis’, ‘response to insulin’) and with their known functions in other tissues as epigenetic regulators and pioneer transcription factors (e.g. ‘chromosome organization’, ‘chromatin remodelling’, ‘regulation of histone methylation’, ‘DNA methylation’, ‘DNA conformational change’) (Fig. 5A, Table S4). Many terms associated with mRNA splicing were also over-represented (Fig. 5A). Genes involved in mRNA splicing showed multiple changes in exon usage, indicating that mRNA splicing factors are themselves subject to alternative splicing in developing T cells, and that the regulatory effects on splicing of the Foxa transcription factors may be amplified by regulation of splicing of components of the splicing machinery (Fig. 5B-D). Additionally, the enrichment analysis highlighted positive regulation of NFKβ signalling, a pathway that has been shown to regulate alternative splicing in T cells (Mallory et al., 2015).

### Foxa1 and Foxa2 regulate alternative splicing of essential genes for T-cell development

**Fig. 6.**
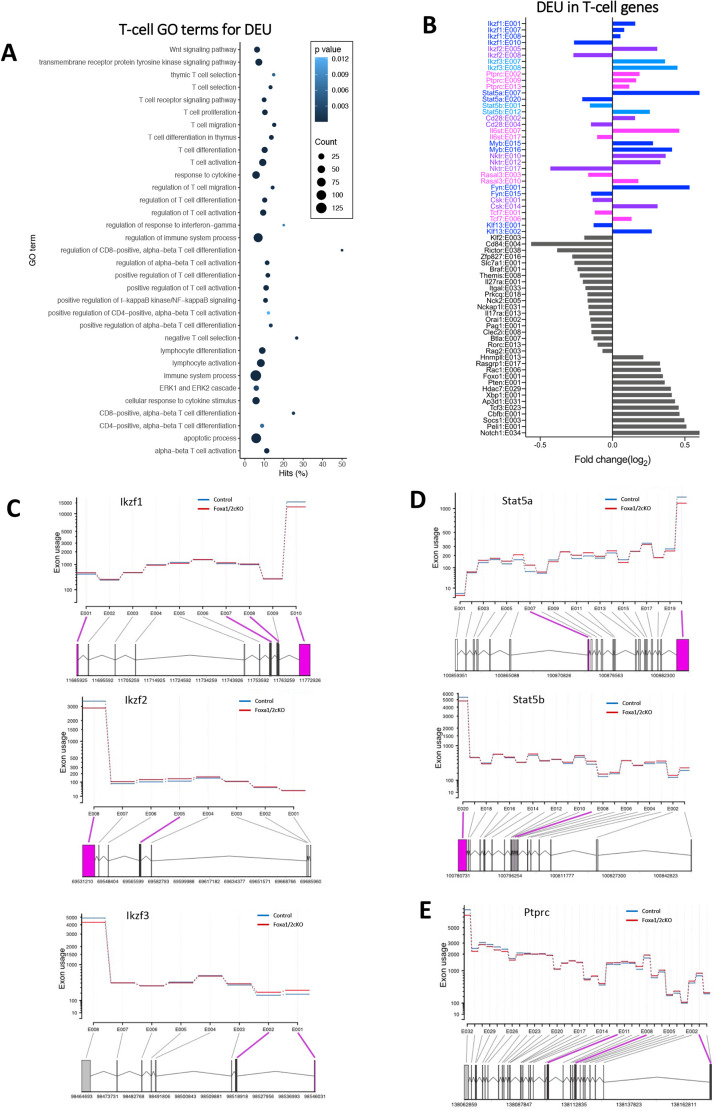
**Foxa1/2 regulates alternative splicing of genes required for T-cell development.** (A) Dot plot of Gene Ontology (GO) analysis for genes showing DEU. Biological processes associated with T-cell development and function are shown. The *y*-axis represents the biological process GO terms and the *x*-axis represents the percentage of genes found in that GO term category. Dot size represents the number of genes and the colour indicates the *P*-value. (B) Bar chart shows genes with DEU that are involved in T-cell development. Genes in colour represent those with multiple differential exon changes; genes in grey have single differential exon changes. (C-E) DEXSeq representations of *Ikzf1*, *Ikzf2*, *Ikzf3* (C), *Stat5a*, *Stat5b* (D) and *Ptprc* (E) that show significant DEU between Foxa1/2 cKO (red) in comparison with control (blue). The exons highlighted in pink have DEU (FDR-adjusted *P*≤0.05).

GO terms associated with T-cell development and function showed over-representation of DEU genes (Fig. 6A), for example ‘thymic T-cell selection’, ‘T-cell receptor signalling pathway’, ‘regulation of CD8-positive αβ differentiation’, ‘lymphocyte differentiation’, ‘CD4-positive alpha-beta T-cell differentiation’. Thus, Foxa1/2 control exon usage for genes involved in processes essential for the transition from DP to SP cell. Multiple differentially used exons were found in key genes for this transition, such as *Ikzf1*, *Ptprc*, *Stat5a*, *Stat5b*, *Cd28* and *Klf13* (Fig. 6B) (Park et al., 2010; Urban and Winandy, 2004; Mee et al., 1999; Watanabe et al., 2020; Outram et al., 2008), and many other essential genes for T-cell development showed differential usage of a single exon (for example, *Notch1*, *Rorc*, *Socs1*, *Pten*, *Orai1*, *Cbfb* and *Ets2*) (Fig. 6B, Table S3). Many of the T-lineage genes that contained significant DEU have been previously described to be alternatively spliced during lymphocyte development or activation, such as the Ikaros family members *Fyn*, *Ptprc* (CD45), *Stat5a*, *Stat5b* and *Cd28* (Martinez and Lynch, 2013; Cho et al., 2014) (Fig. 6B-E). However, the DEU caused by the absence of Foxa1 and Foxa2 did not always correspond to well-described alternatively spliced variants of these genes. For example, alternative splicing of *Ptprc* exons 4, 5 and 6 is functionally important in T-cell differentiation (Cho et al., 2014), but in our datasets, despite variation in expression of exon 5, only exons 2, 9, 13 were significantly different between control and conditional knockout after adjustment for false discovery (*P*<0.05) (Fig. 6B,E). Likewise, for *Ikzf1*, *Ikzf2* and *Ikzf3* we identified differentially expressed exons that were distinct from the alternative splice variants described in mouse thymocytes (Mitchell et al., 2017), indicating that absence of Foxa1 and Foxa2 led to wide dysregulation in the RNA splicing of genes that would normally display splice variants (Fig. 6B,C).

**Fig. 7.**
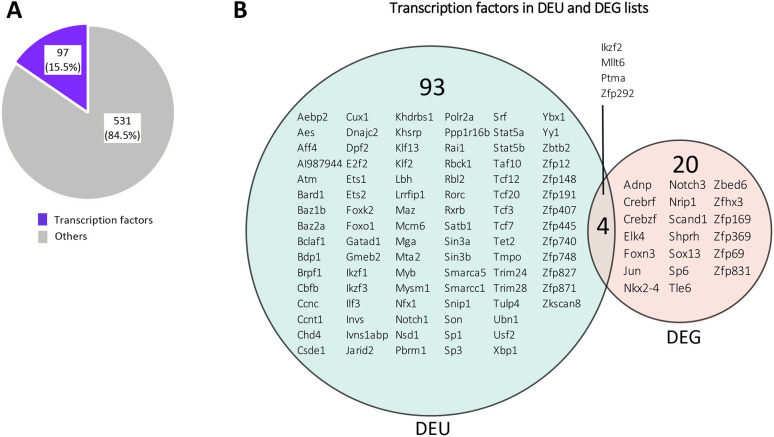
**Foxa1/2 regulates alternative splicing of genes that encode transcription factors required for the transition from DP to SP thymocyte.** (A) Pie chart illustrates the number of genes showing DEU. The purple section represents the number of genes that encode transcription factors, and the grey section represents the number of genes that do not encode transcription factors. (B) Proportional Venn diagram illustrates the intersection between DEGs that encode transcription factors (pink) and genes showing DEU that encode transcription factors (green), listing the genes in each category.

Given the many changes in exon usage observed in genes required for T-cell development and positive selection, it seemed likely that the function of Foxa1/2 during positive selection is to regulate RNA splicing of essential genes for developmental progression. Therefore, to investigate the impact of Foxa1/2 on the normal splicing of regulators of development, we identified transcription factors within the genes that showed DEU between Foxa1/2cKO and control. Of the 628 genes that showed DEU, 97 (15.5%) encoded transcription factors (Fig. 7A, Table S2), many of which are important during T-cell development, and are specifically required for the transition from DP to SP cell (for example, *Yy1*, *Trim28*, *Tcf7*, *Tcf3*, *Tcf12*, *Stat5a*, *Stat5b*, *Sp3*, *Rorc*, *Klf13*, *Cbfb*) (Fig. 7B).

## DISCUSSION

Here, we show that the pioneer transcription factors Foxa1 and Foxa2 regulate alternative RNA splicing during T-cell development at the transition from DP to SP thymocyte. Conditional deletion of Foxa1 and Foxa2 from developing DP thymocytes led to reduced positive selection and a partial arrest at the transition from DP to SP thymocyte, with a reduction in SP cell maturation, and a reduced peripheral naïve CD4 T-cell population. Conditional deletion of Foxa1 and Foxa2 also led to significant changes in the expression of genes that regulate RNA splicing in cells undergoing positive selection, and concomitantly to >850 significantly differentially used exons.

Alternative RNA splicing is a mechanism that enables cells to generate many different proteins from a limited number of genes and is important in the regulation of development processes, including T-cell development (Sznajder et al., 2020; Mallory et al., 2015; Martinez and Lynch, 2013; Baralle and Giudice, 2017; Wu et al., 2010). Our study indicates that Foxa1 and Foxa2 regulate the mRNA splicing of many genes that are important for progression at this developmental transition, and we therefore propose that aberrant RNA splicing of multiple genes may account at least in part for the reduction in positive selection and differentiation to SP cell observed when Foxa1 and Foxa2 were conditionally deleted from DP cells. In support of this, many of the genes that showed DEU, but were not DEGs, between Foxa1/2cKO and control datasets are well-known to be required at this transition (e.g. *Socs1*, *Ptprc*, *Rasgrp1*), and many encode transcriptional regulators of this stage of development (e.g. *Ikzf1*, *Stat5a*, *Stat5b*, *Cbfb*, *Tcf7*, *Tcf3*, *Tcf12*, *Klf13*, *Sp3*, *Rorc*)*.* GO term enrichment analysis also highlighted terms associated with positive selection and differentiation to SP. In contrast, few DEGs encoded known relevant transcription factors or regulators of TCR repertoire selection or differentiation from DP to SP, and *Themis*, a DEG with a verified Foxa1/2-binding site, which was more highly expressed in the conditional knockout, and which can modulate TCR signalling in thymocytes, also showed DEU. The way in which the transcriptional activity of Foxa1/2 relate to their regulation of splicing will require further investigation, as there is increasing evidence that splicing can occur co-transcriptionally as well as post-transcriptionally (Tellier et al., 2020). Interestingly, approximately one-third of genes that showed DEU but were not DEGs had previously been shown to bind directly to Foxa1/2 in neuronal progenitors, suggesting that Foxa1/2 may act directly to regulate their splicing.

Overall, we identified only 176 DEG between Foxa1/2cKO and control CD69^+^DP cells, and of these >60% were more highly expressed in the conditional knockout than control, suggesting either that they were directly repressed by Foxa1/2, most likely by association of Foxa1/2 with a co-repressor, or that Foxa1/2 activate the transcription of an intermediate transcriptional repressor. Approximately 48% of DEGs have previously been shown to bind Foxa1/2 in whole-genome ChipSeq screen of neuronal progenitors (Metzakopian et al., 2012, 2015), suggesting that they were also likely to be direct targets (directly bound by Foxa1/2) in developing T cells. These were distributed between DEGs that were up- or downregulated in the Foxa1/2cKO compared with control, and ∼51% of upregulated genes have previously been shown to bind Foxa1/2 by ChipSeq screening in other cell types (Metzakopian et al., 2012, 2015). We therefore think it is most likely that absence of Foxa1/2 in CD69^+^DP cells led to increased expression of these DEGs because of a direct repressive impact of Foxa1/2 binding to these sites and recruitment of a co-repressor in control cells, rather than indirectly by transcriptional activation of an unknown downstream repressor of transcription. Although in cell transfection assays Foxa transcription factors behave as transcriptional activators, Foxa1/2 have previously been shown to have repressor activity in other developing tissues, and Foxa2 has been shown to interact with the transcriptional co-repressor Tle family of proteins (Metzakopian et al., 2012; Sekiya and Zaret, 2007; Wang et al., 2000). Further investigation of Foxa2-Tle interactions in the regulation of differentiation from DP to SP cell will therefore be important, given that Tle1, Tle3 and Tle4 are together required for commitment to the CD8 lineage at the transition from DP to SP (Xing et al., 2018).

Foxa1/2 conditional deletion led to increased transcription of several RNA-splicing regulators, including *Sf3b1*, *Smg1* and *Mbnl1*, mutations of which are associated with haematological malignancies and aberrant RNA splicing, but also significantly decreased transcription of splicing factors (*Prpf40b*, *Hnrnpa1*, *Snrpd3*) and the histone linker *H1f0*, which may be required for mRNA splice site recognition (Kalashnikova et al., 2013; Ho et al., 2004; Wang et al., 2012; Sznajder et al., 2020; Itskovich et al., 2020; Shiozawa et al., 2018; Mupo et al., 2017; McIlwain et al., 2010; Du et al., 2014). Overall loss of Foxa1 and Foxa2 led to broad changes in splicing events, indicating that Foxa1 and Foxa2 are important regulators of mRNA processing during T-cell development. Foxa1/2 also regulated the mRNA of many splicing genes that encode splicing factors, suggesting that their influence on the splicing machinery is further amplified by regulation of alternative splicing of splicing components, as well as by a more direct effect on the transcription of splicing regulators, RNA binding and histone linker proteins.

Mbnl1 is highly expressed in the thymus and a recent study demonstrated that its constitutive knock out led to a hyperplastic thymus with retention of thymocytes and many mis-splicing events (Sznajder et al., 2020). *Mbnl1* was upregulated approximately two-fold in CD69^+^DP cells in the absence of Foxa1 and Foxa2 and was one of the most significantly differentially expressed genes in our datasets. In Foxa1/2cKO DP thymocytes, we observed DEU in some of the same genes that were affected when *Mbnl1* was constitutively deleted: for example, *Tcf7* exon 10 was affected by absence of Mbnl1, whereas our datasets showed changes in *Tcf7* exons 1 and 6; *Map4k4* exon 20 was affected in Mbnl1^−/−^ thymus, but Foxa1/2cKO led to changes in *Map4k4* exons 15 and 33; *Sptan1* exon 23 was affected in Mbnl1^−/−^ thymus, but Foxa1/2cKO changed usage of *Sptan1* exons 2, 11, 12 and 49 (Sznajder et al., 2020).

Conditional deletion of Foxa1 alone from DP thymocytes did not grossly affect αβT-cell development, and the double Foxa1/2cKO had a stronger impact on differentiation from DP to SP than the single Foxa2cKO, suggesting overlapping or redundant functions, so that although Foxa1 may not be required at this developmental transition it can partially replace the requirement for Foxa2. Foxa1 and Foxa2 have compensatory and partially redundant roles in other tissues, including liver (Kaestner, 2010), and a recent study showed that conditional deletion of Foxa3 in addition to Foxa1 and Foxa2 in adult liver abrogated liver gene regulatory networks, destroying liver tissue homeostasis and function in the adult (Reizel et al., 2020). It will therefore be important in the future to investigate the potential compensatory role of Foxa3 in T-cell development.

## MATERIALS AND METHODS

### Mice

CD4-Cre transgenic mice (Westendorf et al., 2016) were purchased from The Jackson Laboratory. Foxa1^flox/flox^ Foxa2^flox/flox^ mice were provided by Dr Siew-Lan Ang (Ferri et al., 2007). These mice were backcrossed to C57BL/6 strain for at least six generations. C57BL/6 mice were purchased from Envigo (UK). All mice were bred and maintained at University College London (UK) under UK Home Office regulations.

To delete Foxa1 and/or Foxa2 selectively in T cells, we crossed Foxa1^flox/flox^ and Foxa2^flox/flox^ mice with CD4-cre^+^ mice. Subsequently, Foxa1^flox/flox^ Foxa2^flox/flox^ CD4-cre^+^ mice were bred. In this paper, we refer to these mice as Foxa1/2cKO. Similarly, the Foxa1^flox/flox^ CD4-cre^+^ is termed Foxa1cKO and the Foxa2^flox/flox^ CD4-cre^+^ is termed Foxa2cKO. For all experiments, their control genotype is the CD4-cre^−^ littermate with the same floxed alleles as the experimental animals.

### Genotyping

DNA from mice was extracted by incubation at 56°C overnight in lysis buffer containing 50 mM KCl, 10 mM Tris-HCl (pH 8.5), 1.5 mM MgCl_2_, 0.01% gelatin, 0.45% Nonidet P-40, 0.45% Tween 20 and 0.5 µg/ml Protinease K in ultra-pure water. PCR analysis was carried out as described (Lau et al., 2012). Primer sequences for PCR to detect the *CD4-cre* transgene were: forward 5′-CGATGCAACGAGTGATGAGG-3′, reverse 5′-GCATTGCTGTCACTTGGTCGT-3′.

The PCR conditions for the CD4-cre transgene were: 1 min at 94°C, 1 min at 61°C, and 1 min at 72°C for 35 cycles. *Foxa1* and *Foxa2* WT and floxed gene primers and PCR conditions were as described (Lau et al., 2018).

### Cell sorting

To obtain DN3 (CD4^−^CD8^−^CD25^+^CD44^−^), DN4 (CD4^−^CD8^−^CD25^−^CD44^−^), DP (CD4^+^CD8^+^), CD4SP (CD4^+^CD8^−^CD3^+^), CD8SP (CD4^−^CD8^+^CD3^+^) and CD69^+^DP (CD4^+^CD8^+^CD69^+^), thymocyte cell suspensions were sorted using Molecular Flow Cytometer (Cytomation).

### Quantitative RT-PCR

RNA was extracted using Stratagene StrataPrep Total RNA miniprep kit according to the manufacturer's protocol and cDNA was synthesized using SuperScript III kit (Invitrogen) according to the manufacturer's protocol. RT-PCR was carried out by analysis of cDNA sample in triplicate on an iCycler (Bio-Rad Laboratories) using the iQ SYBR Green Supermix according to the manufacturer's instructions. The housekeeping gene *Gapdh* was used to allow quantification of template and normalization of each gene*. Gapdh*, *Foxa1* and *Foxa2* RT-PCR primers were purchased from Qiagen (Quantitect primer assay).

### Antibodies and flow cytometry

Cells were stained using combinations of the following directly conjugated antibodies supplied by BioLegend or eBioscience: anti-CD3, anti-CD4, anti-CD5, anti-CD8, anti-CD24, anti-CD44, anti-CD62L, anti-CD69, anti-Foxp3, anti-NK1.1, anti-Qa2 anti-Nr4a1 and anti-TCRβ (Table S5). Cell suspensions were stained for 30 min on ice in PBS supplemented with 2% fetal calf serum and 0.01% sodium azide. For intracellular Foxp3 and Nr4a1 staining, cells were fixed and permeabilized using the Intracellular Fixation & Permeabilization kit (eBioscience). AnnexinV staining was carried out as described (Solanki et al., 2020). Samples were analysed on an Accuri C6 flow cytometer (BD Biosciences) and LSRII (BD Biosciences). Data were analysed using FlowJo 10.4.2 (Tree Star).

### RNA sequencing and data analysis

CD69^+^CD4^+^CD8^+^ thymocytes from Foxa1/2cKO and control thymus were FACS sorted. Each sample was sorted independently from different mice, with two mice of each genotype per sort. RNA was prepared as described (Solanki et al., 2017) and sequenced by UCL Genomics on the Illumina Next Seq 500. The BaseSpace Sequence Hub was used for both FASTQ generation and RNA-Seq alignment to the mouse reference genome UCSC mm10 (RefSeq gene annotation). Aligned reads were counted using the HTSeq python package with ‘union’ overlap resolution mode (Anders et al., 2015). The Bioconductor package DESeq2 (1.30.0) was used to test for differential expression (Love et al., 2014). *P*-values were plotted in a histogram resulting in a hill-shape, indicating an overestimation of the variance in the null distribution (Fig. S1). Therefore, the *z*-scores returned by DESeq2 were used as input to the CRAN package fdrtool (1.2.16) to re-estimate the null variance and subsequently the *P*-values (Strimmer, 2008). Adjusted *P*-values were then calculated by Benjamini–Hochberg false discovery correction (5%). Genes with adjusted *P*-values <0.05 were considered as DEGs.

The Bioconductor package DEXSeq (1.36.0) was used to test for DEU (Anders et al., 2012; Reyes et al., 2013). The Python script dexseq_prepare_annotation.py was used to prepare the genome annotation with the following parameter: ‘-r no’. Subsequently, dexseq_count.py was used to generate counts of exons using the BAM alignment files as input with the following parameters: ‘-p yes -s no -a 0’. The exon count files were then inputted into DEXSeq. Adjusted *P*-values were then calculated by Benjamini–Hochberg false discovery correction (5%).

To identify over-represented gene ontology terms in the set of DEGs and genes with DEU, we used the Bioconductor package GOseq (1.42.0) (Young et al., 2010). For data visualization, we used regularized logarithm (rlog)-transformed counts generated by DESeq2 as input for heatmaps, which were generated using the CRAN package pheatmap (1.0.12): rows were centred; unit variance scaling was applied to rows; and both rows and columns were clustered using Pearson correlation distance and average linkage, where red represents higher expression and blue lower expression on a linear correlation scale. A value of 1 indicates a positive association, a value of −1 indicates a negative association, and a value of 0 indicates no association. Venn diagrams were generated using the CRAN package VennDiagram (1.6.20).

To identify verified transcription factors among DEGs and DEU genes, we merged the most recent updated lists from the Riken mouse transcription factor database (Kanamori et al., 2004) and the mouse transcription factor list from The Animal Transcription Factor DataBase (Hu et al., 2019) and intersected our gene lists with this merged list.

Canonical correspondence analysis is a multivariate analysis that allows the comparison of experimental transcriptome data with publicly available datasets from other laboratories (Ono et al., 2014). CCA was performed using the CCA function of CRAN package vegan, as previously described (Solanki et al., 2020). The GSE38909 dataset was used as the environmental variable and our dataset was regressed onto it. The GSE38909 dataset contains DP thymocytes from AND-TCR transgenic mice stimulated with a positively selecting peptide (gp250) or a non-selecting control peptide Hb (Lo et al., 2012). To represent environmental variables of interest, the 2000 most significant DEGs (lowest *P*-values, calculated by moderated eBayes adjusted for false positives) between DP thymocytes stimulated with a non-selecting control peptide and DP thymocytes stimulated with the positively selecting peptide were used to generate a scale of unstimulated to TCR-signalling-for-positive-selection, and we regressed our datasets onto this axis.

## Supplementary Material

Supplementary information
