# Supplementary material for: The pioneer transcription factors Foxa1 and Foxa2 regulate alternative RNA splicing during thymocyte positive selection
Source: Development. 2021 Jul 29;148(15):dev199754. doi: 10.1242/dev.199754 (PMC8353164; doi:10.1242/dev.199754)
Supplement: Supplementary information [file develop-148-199754-s1.pdf]

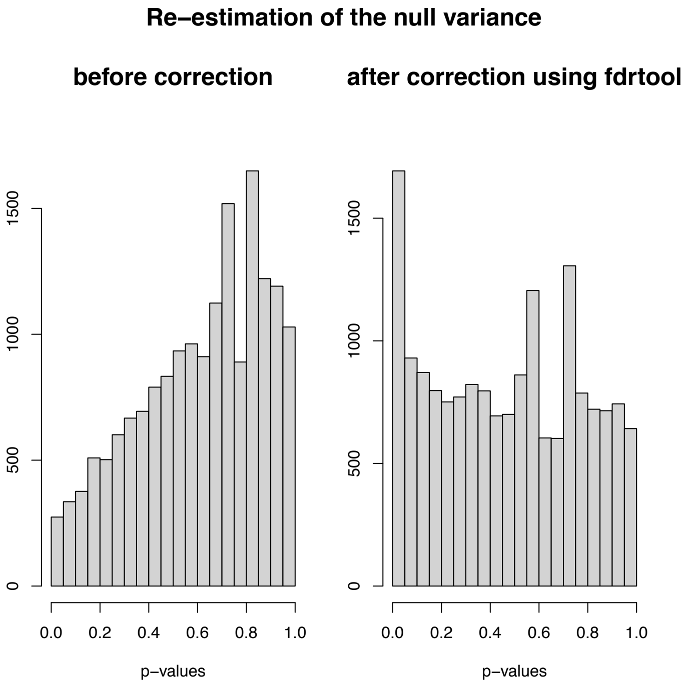

Fig. S1. P-value histogram showing before and after correction of overestimation of variance. Left, histogram of P-values obtained using DeSeq2 showing a hill-shape, indicating an overestimation of the variance in the null distribution. Right, histogram of P-values after z-scores returned by DESeq2 were used as input to fdrtool to re-estimate the null variance.

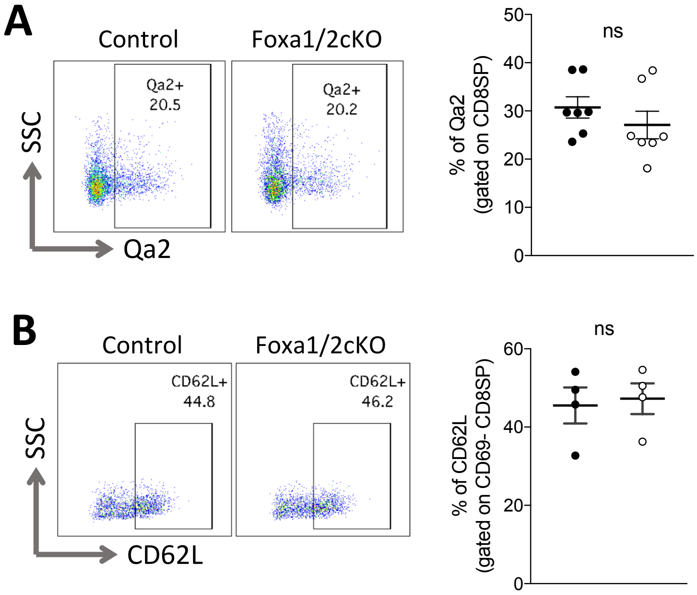

Fig. S2. (A) Flow cytometry profiles show anti-Qa2 staining, gated on CD8SP, giving the percentage of cells in the region shown. Scatter plot shows percentage of $Qa2^{+}$ cells in the CD8SP population. **(B)** Flow cytometry profiles show anti-CD62L staining, gated on the $CD69^{-}$ CD8SP population. Scatter plot shows the percentage of $CD62L^{+}$ cells in the $CD69^{-}$CD8SP population.

Table S1. RNA-Seq datasets from CD4+CD8+CD69+ thymocytes between control and Foxa1/2cKO.
List of DEG (adjusted p-value < 0.05).
Gene names highlighted in blue indicate genes found in Foxa2 targets in (34).
Gene names highlighted in pink indicate genes found in Foxa1 targets in (44).
Gene names highlighted in purple indicate genes found in both Foxa1 and Foxa2 targets in (34, 44).
Gene names in green indicate genes transcriptionally regulated during positive selection (45).

Click here to download Table S1

Table S2. Biological Processes(BP) GO term enrichment analysis of DEG GO analysis of biological processes associated with 176 DEG.

Click here to download Table S2

Table S3. Differential exon usage analysis by DEXSeq from CD4+CD8+CD69+ thymocytes between control and Foxa1/2cKO.
List of the DEU (adjusted p-value < 0.05).
*CD4-Cre transgene contains first 2 exons of CD4 (Exon9 and 10 on reverse strand)(94)
Gene names highlighted in blue indicate genes found in Foxa2 targets in (34).
Gene names highlighted in pink indicate genes found in Foxa1 targets in (44).
Gene names highlighted in purple indicate genes found in both Foxa1 and Foxa2 targets in (34, 44).

Click here to download Table S3

Table S4. Biological Processes(BP) GO term enrichment analysis of DEU
GO analysis of biological processes associated with genes containing DEU.
padj = p-value after Benjamini-Hochberg false discovery correction

Click here to download Table S4

Table S5. List of antibodies used in the study

Antibody	Fluorochrome	Catalogue number	Company
CD3	Brilliant Violet 421	100227	Biolegend
CD3	Alexa Fluor® 700	100215	Biolegend
CD4	APC/Cyanine7	100413	Biolegend
CD4	PerCP/Cyanine5.5	100433	Biolegend
CD5	PerCP/Cyanine5.5	100623	Biolegend
CD8	Brilliant Violet 421	100737	Biolegend
CD8	PE	100707	Biolegend
CD24	Brilliant Violet 605	101827	Biolegend
CD44	Brilliant Violet 421	103039	Biolegend
CD62L	APC	104411	Biolegend
CD69	APC/Cyanine7	104525	Biolegend
Foxp3	Alexa Fluor® 647	126407	Biolegend
NK1.1	PE/Cyanine7	156513	Biolegend
Qa2	FITC	121709	Biolegend
Nr4a1	PE	12-5965-82	eBioscience
TCRb	PE/Cyanine7	109221	Biolegend
